# Supplementary figures and images for: Determination of sulfamerazine in aquatic products by molecularly imprinted capillary electrochromatography
Source: R Soc Open Sci. 2019 Jun 26;6(6):190119. doi: 10.1098/rsos.190119 (PMC6599784; doi:10.1098/rsos.190119)

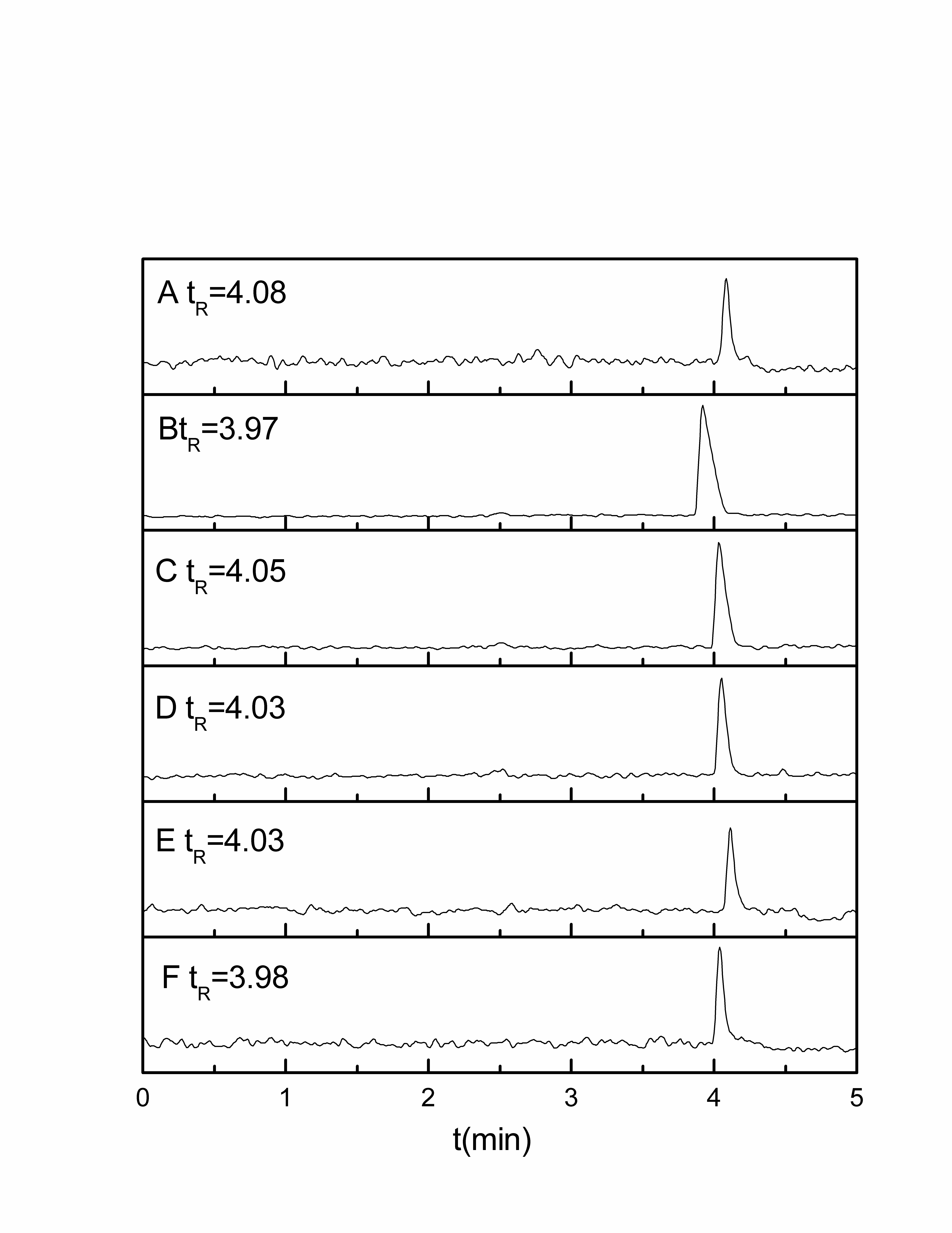

Supplement: The reproducibility research of molecularly imprinted monolith [file rsos190119supp1.jpg]

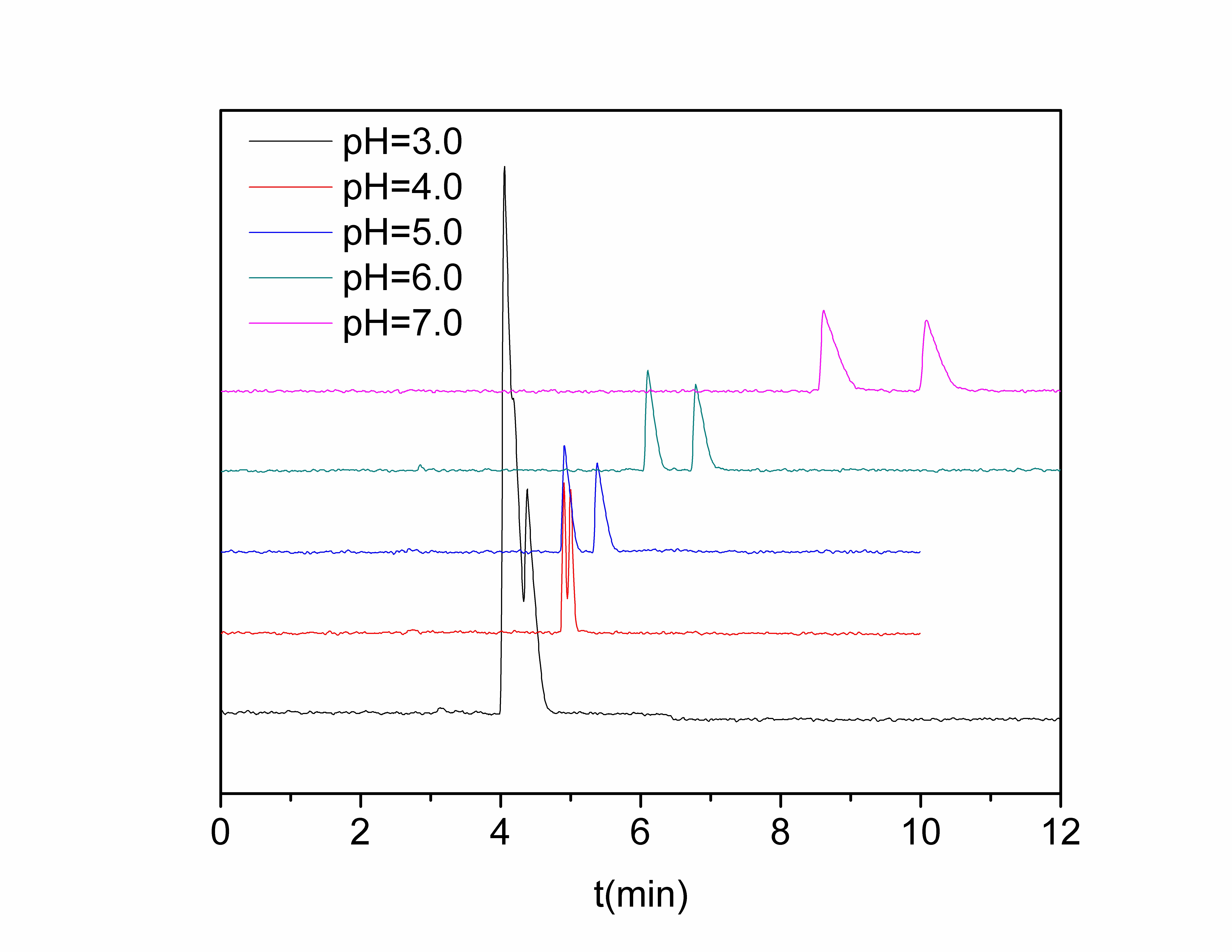

Supplement: The separative capacity of molecularly imprinted monolith [file rsos190119supp2.jpg]

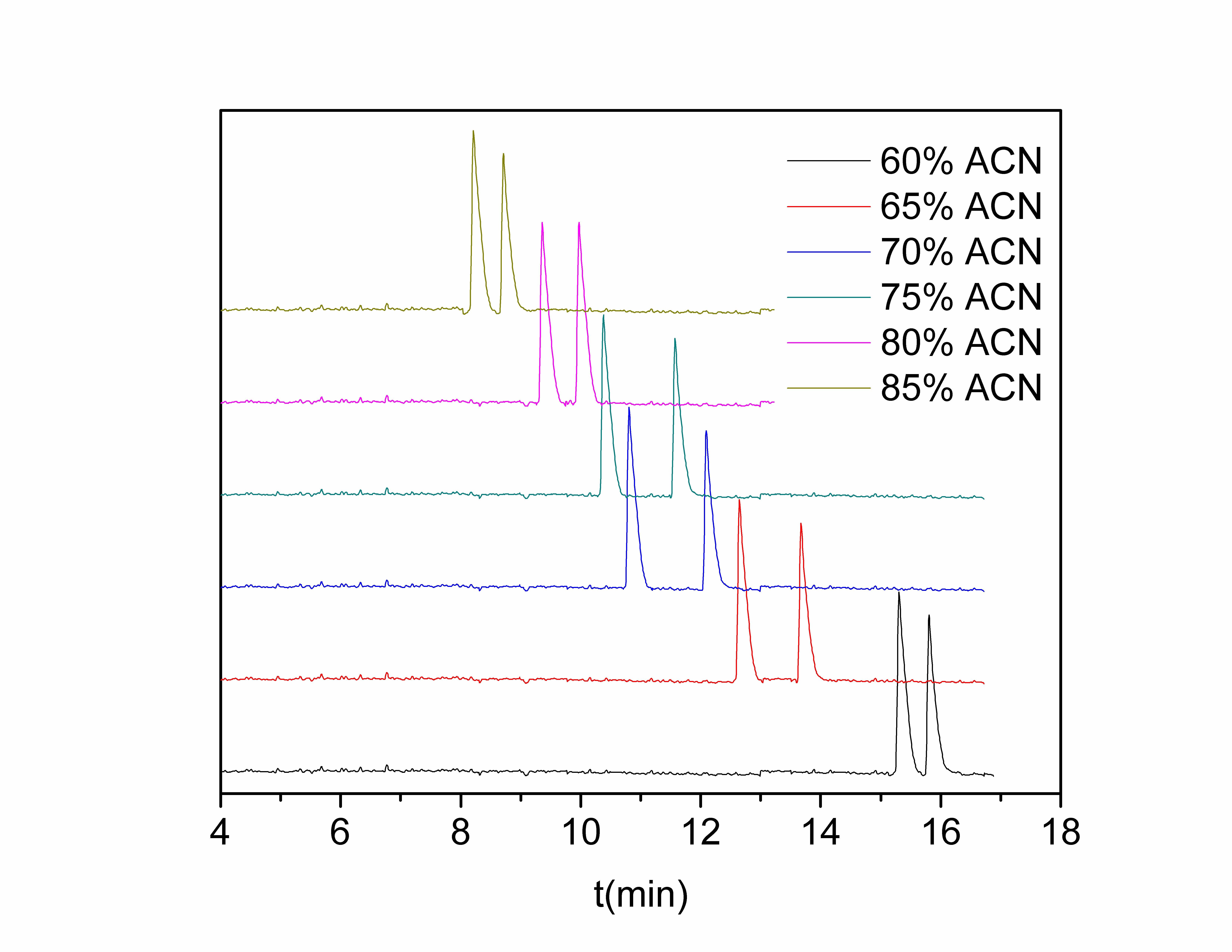

Supplement: The separative capacity of molecularly imprinted monolith [file rsos190119supp3.jpg]

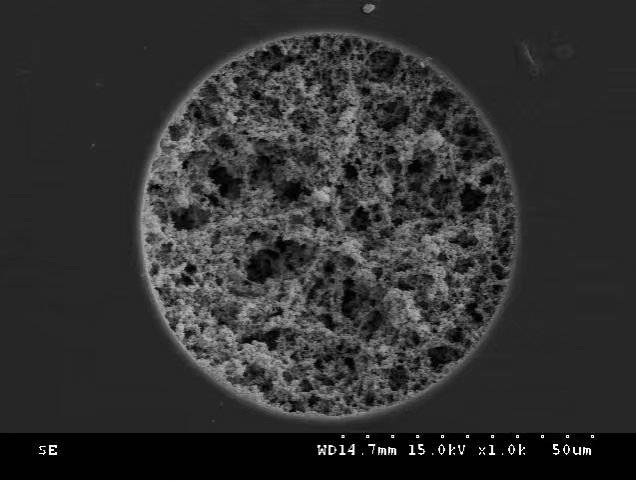

Supplement: The intersecting surface of molecularly imprinted monolith [file rsos190119supp4.jpg]
